# Association of erlotinib and acitretin for the treatment of Olmsted syndrome with erythromelalgia in a pediatric patient^☆^

**DOI:** 10.1016/j.abd.2026.501313

**Published:** 2026-03-26

**Authors:** Luna Azulay-Abulafia, Joice Rodrigues Fagundes, Edgar Efren Ollague Cordova, Karla Diniz Pacheco, Alain Hovnanian

**Affiliations:** a Instituto de Dermatologia Professor Rubem David Azulay, Santa Casa da Misericórdia do Rio de Janeiro, Rio de Janeiro, RJ, Brazil; b INSERM UMR1163, Laboratory of Genetic Skin Diseases, Imagine Institute of Genetic Diseases, Université Paris Cité, Paris, France

Dear Editor,

Olmsted Syndrome (OS) is a rare genodermatosis characterized by painful and mutilating Palmoplantar Keratoderma (PPK) and periorificial keratotic plaques. It is an orphan disease, which represents a therapeutic challenge. OS is most often caused by dominant gain-of-function pathogenic variants in the TRPV3 gene. We report an 8-year-old male suffering from OS with erythromelalgia, who was treated since the age of 18-months with acitretin and topicals, leading to only minor improvement. At the age of 5-years, oral erlotinib was added to acitretin, resulting in a significant improvement in pain, keratoderma and erythromelalgia, with sustained efficacy and safety.

The patient is a full-term male newborn from non-consanguineous parents with no family history of skin disease. From the age of 5-months, the patient developed painful and pruritic PPK. Over time, PPK progressed bilaterally and was associated with periorificial hyperkeratosis. Upon physical examination, the patient presented with asymmetric, thick, and localized or diffuse bilateral PPK (Fig. 1A), keratotic plaques around the anus, ears, and mouth alongside leukokeratosis on the tongue (Fig. 1B). The scalp hair was fine and reduced. Congenital left-sided clubfoot was additionally observed.

**Fig. 1:**
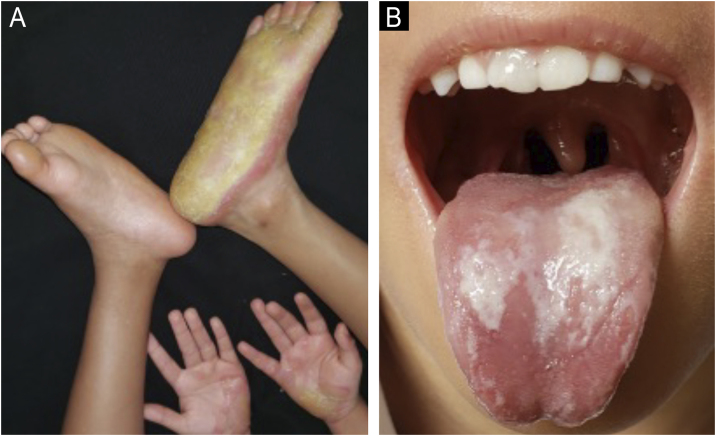
(A) Erythema, calluses and hyperkeratotic palmoplantar lesions under treatment using acitretin only. (B) Keratotic plaques around the mouth alongside leukokeratosis on the tongue using acitretin only.

Genomic DNA was extracted from the patient’s peripheral blood for genetic testing. Next-generation sequencing analysis using a custom gene panel revealed a heterozygous c.1703>T; p.GIy568VaI pathogenic missense variant in the TRPV3 gene. The parents did not carry this variant.

Topical treatments (0.1% retinoic acid cream, 20% urea cream, corticosteroids and emollients) were ineffective. Acitretin treatment (1 mg/kg/day) was started at 18-months, resulting in moderate improvement of the calluses, but with very limited effect on pain.

Intense burning pain was associated with itching, redness and warmth, triggered by heat and relieved by cooling in the extremities, a typical presentation of erythromelalgia. The patient was therefore referred to the pain clinic and a treatment with gabapentin and tramadol was associated with acitretin, which only partially reduced pain.

Due to persistent symptoms at the age of 4-years, oral erlotinib (50 mg/day, alternating with 25 mg/day) (his weight was 13 kg), was added to acitretin. The association resulted in a significant improvement in pain, pruritus, sleep, mobility, reduced irritability, analgesic use, disappearance of erythromelalgia attacks and a significant improvement in PPK (Fig. 2, Fig. 3), keratotic plaques in the perioral region and leukokeratosis on the tongue within four weeks of initiation (Fig. 4). After 52-months of combined treatment, the child had no adverse events and gained weight.

**Fig. 2:**
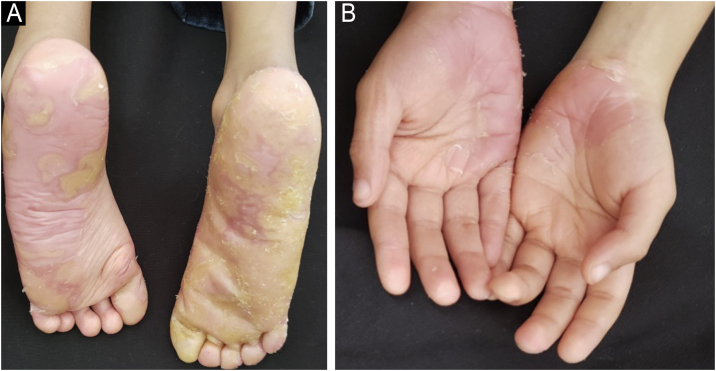
(A and B) Treatment with acitretin and systemic erlotinib for 16-months reduced painful PPK and erythema.

**Fig. 3:**
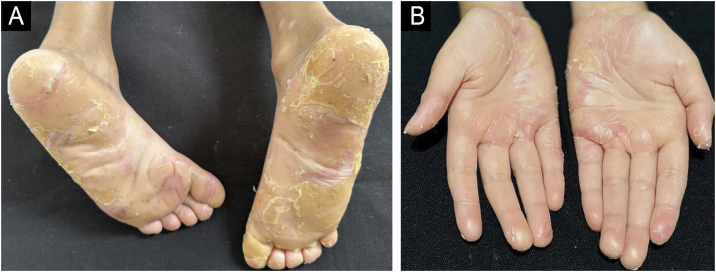
(A and B) Aspect at 46-months of the combined treatment showing reduced PPK and no erythema.

**Fig. 4:**
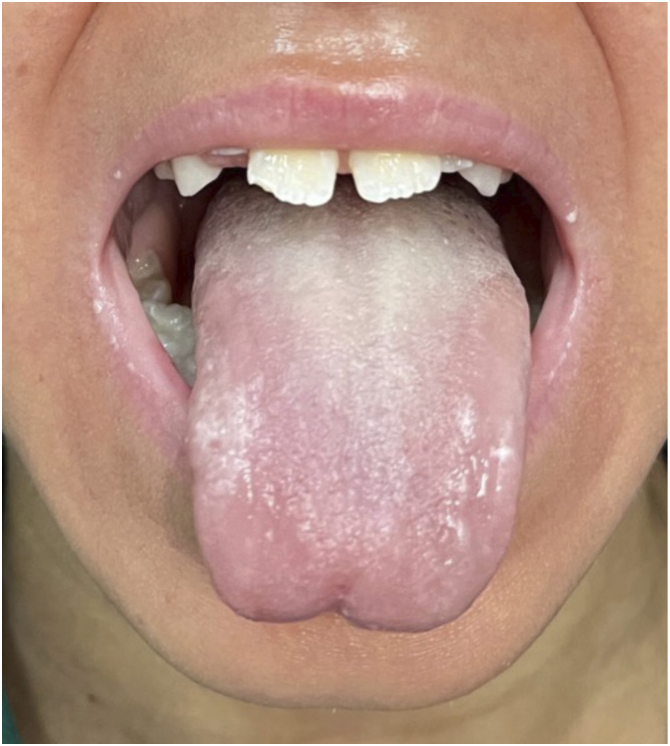
Current aspect at 52-months of treatment showing absence of keratotic plaques around the mouth and absence of leukokeratosis on the tongue.

This is the fourth reported case with the p.GIy568VaI mutation in TRPV3.^1^, ^2^ The Asian patients carrying this mutation presented with severe disease; another Brazilian patient had focal keratoderma.^1^, ^2^ These reports did not mention erythromelalgia, indicating clinical variability for the same mutation.

TRPV3 encodes a non-selective tetrameric cationic channel predominantly expressed in keratinocytes and sensory neurons, activated by temperature (31‒39 °C) or chemical ligands. It is implicated in skin inflammation and nociceptive signalling, and is involved in erythromelalgia. In eight previously reported OS patients, erythromelalgia has been described, suggesting that it is not a coincidental feature of TRPV3-related OS.^1^, ^2^, ^3^

Currently, there is no specific treatment for OS, although the repurposing of erlotinib for this condition has shown an important benefit, as in this case. Erlotinib is an Epidermal Growth Factor Receptor (EGFR) tyrosine kinase inhibitor approved for the treatment of non-small cell lung cancer and advanced pancreatic cancer. This drug inhibits the EGFR, affecting both wild-type and mutated EGFRs with EGFR mutations, which are essential for cellular differentiation, proliferation, and angiogenesis. Studies have shown that the EGFR pathway is activated in OS and that oral EGFR inhibitor (erlotinib) has a drastic effect on the reduction of pain and keratoderma in OS patients.^4^, ^5^, ^6^

We report efficacy of the combination of erlotinib and acitretin in a child with severe OS and erythromelalgia, who showed a dramatic positive response in pain symptoms and significant reduction of keratoderma. Changes in hair and eyelashes (such as trichomegaly, curling, or increased fragility) are recognized cutaneous adverse effects of erlotinib. In the present case, these alterations were not observed during treatment.

Patient improvement has been sustained for more than 52-months with no side effects, indicating that combined treatment of erlotinib and acitretin can be effective and well tolerated for a long period in OS with erythromelalgia.

## ORCID ID

Luna Azulay-Abulafia: 0000-0002-4698-2009

Joice Rodrigues Fagundes: 0000-0003-0436-5261

Edgar Efren Ollague Cordova: 0000-0001-9180-3527

Karla Diniz Pacheco: 0009-0008-8836-1391

Alain Hovnanian: 0000-0003-3412-7512

## Financial support

None declared.

## Authors’ contributions

Luna Azulay-Abulafia: Conceptualization; methodology; writing-original draft.

Joice Rodrigues Fagundes: Data collection; investigation; writing-review & editing.

Edgar Efren Ollague Cordova: Formal analysis; visualization; writing-review & editing.

Karla Diniz Pacheco: Supervision; Project administration; writing-review & editing.

Alain Hovnanian: Validation; Resources; writing-review & editing.

## Research data availability

Does not apply.

## Conflicts of interest

None declared.
